# Cluster and propensity based approximation of a network

**DOI:** 10.1186/1752-0509-7-21

**Published:** 2013-03-14

**Authors:** John Michael Ranola, Peter Langfelder, Kenneth Lange, Steve Horvath

**Affiliations:** 1 Biomathematics, University of California, Los Angeles, CA, USA; 2 Human Genetics, UCLA, Los Angeles, CA, USA; 3 Biostatistics, UCLA, Los Angeles, CA, USA; 4 Statistics, UCLA, Los Angeles, CA, USA

**Keywords:** Network decomposition, Model-based clustering, MM algorithm, Propensity, Network conformity

## Abstract

**Background:**

The models in this article generalize current models for both correlation networks and multigraph networks. Correlation networks are widely applied in genomics research. In contrast to general networks, it is straightforward to test the statistical significance of an edge in a correlation network. It is also easy to decompose the underlying correlation matrix and generate informative network statistics such as the module eigenvector. However, correlation networks only capture the connections between numeric variables. An open question is whether one can find suitable decompositions of the similarity measures employed in constructing general networks. Multigraph networks are attractive because they support likelihood based inference. Unfortunately, it is unclear how to adjust current statistical methods to detect the clusters inherent in many data sets.

**Results:**

Here we present an intuitive and parsimonious parametrization of a general similarity measure such as a network adjacency matrix. The cluster and propensity based approximation (CPBA) of a network not only generalizes correlation network methods but also multigraph methods. In particular, it gives rise to a novel and more realistic multigraph model that accounts for clustering and provides likelihood based tests for assessing the significance of an edge after controlling for clustering. We present a novel Majorization-Minimization (MM) algorithm for estimating the parameters of the CPBA. To illustrate the practical utility of the CPBA of a network, we apply it to gene expression data and to a bi-partite network model for diseases and disease genes from the Online Mendelian Inheritance in Man (OMIM).

**Conclusions:**

The CPBA of a network is theoretically appealing since a) it generalizes correlation and multigraph network methods, b) it improves likelihood based significance tests for edge counts, c) it directly models higher-order relationships between clusters, and d) it suggests novel clustering algorithms. The CPBA of a network is implemented in Fortran 95 and bundled in the freely available R package PropClust.

## Background

The research of this article was originally motivated by two types of network models: correlation networks and multigraphs. After reviewing these special network models, we describe how structural insights gained from them can be used to tackle research questions arising in the study of general networks specified by network adjacencies and more generally to unsupervised learning scenarios modeled by similarity measures.

### Background: adjacency matrix and multigraphs

Networks are used to describe the pairwise relationships between *n* nodes (or vertices). For example, we use networks to describe the functional relationships between *n* genes. We consider networks that are fully specified by an *n* × *n ***adjacency matrix *A*** = (*A*_*ij*_), whose entry *A*_*ij*_ quantifies the connection strength from node *i* to node *j*. For an **unweighted** network, *A*_*ij*_ equals 1 or 0, depending on whether a connection (or link or edge) exists from node *i* to node *j*.

For a **weighted network**, *A*_*ij*_ equals a real number between 0 and 1 specifying the connection strength from node *i* to node *j*. For an undirected network, the connection strength *A*_*ij*_ from *i* to *j* equals the connection strength *A*_*ji*_ from *j* to *i*. In other words, the adjacency matrix ***A*** is symmetric. For a directed network, the adjacency matrix is typically not symmetric. Unless we explicitly mention otherwise, we will deal with undirected networks. In this paper the diagonal entries *A*_*ii*_ of the adjacency matrix ***A*** have no special meaning. We arbitrarily set them equal to 1 (the maximum adjacency value); other authors set them equal to 0 [1].

In an **(unweighted) multigraph**, the adjacencies *A*_*ij*_ = *n*_*ij*_ are integers specifying the number of edges between two nodes. A general similarity matrix (whose entries are non-negative real numbers possibly outside [0,1]) can be interpreted as a **weighted multigraph**. In each of the network types, the connectivities

(1) $$\begin{array}{ll} k_{i}= & \underset{j\neq i}{\sum }A_{\text{ij}}\; \end{array}$$

are important statistics pertinent to finding highly connected hubs. In an unweighted network (a graph), *k*_*i*_ is the degree of node *i*.

### Background: correlation- and co-expression networks

Network methods are frequently used to analyze experiments recording levels of transcribed messenger RNA. The gene expression profiles collected across samples can be highly correlated and form modules (clusters) corresponding to protein complexes, organelles, cell types, and so forth [2-4]. It is natural to describe these pairwise correlations in network language. The intense interest in co-expression networks has elicited a number of new models and statistical methods for data analysis [3,5-11], with recent applied research focusing on differential network analysis and regulatory dysfunction [12,13].

A correlation network is a network whose adjacency matrix ***A*** = (*A*_*ij*_) is constructed from the correlations between quantitative measurements summarized in an *m* × *n* data matrix ***X*** = (*x*_*ij*_). The *m* rows of ***X*** correspond to sample measurements (subjects), and the *n* columns of ***X*** correspond to network nodes (genes). The *j*th column ***x***_*j*_ of ***X*** serves as a **node profile** across the *m* samples. A correlation network adjacency matrix is constructed from the pairwise correlations Corr(***x***_*i*_,***x***_*j*_) in either of two ways. An unweighted gene co-expression network is defined by thresholding the absolute values of the correlation matrix. A weighted adjacency matrix is a continuous transformation of the correlation matrix. For reasons explained in [5,14], it is advantageous to define the adjacency *A*_*ij*_ between two genes *i* and *j* as a power *β* ≥ 1 of the absolute value of their correlation coefficient; thus,

$$\begin{array}{lll} A_{\text{ij}}= & |\text{Corr}(x_{i},x_{j}){|}^{\beta }.\; & \; \end{array}$$

Weighted gene co-expression networks have found many important medical applications, including identifying brain cancer genes [14], characterizing obesity genes [15,16], understanding atherosclerosis [17], and locating the differences between human and chimpanzee brains [9]. One of the important steps of weighted correlation network analysis is to find network modules, usually via hierarchical clustering. Each module (cluster) is then represented by the module eigengene defined by a certain singular value decomposition (SVD). Suppose ***Y*** denotes the expression data of a single module (cluster) after the appropriate columns of ***X*** have been extracted and standardized to have mean 0 and variance 1. The SVD of ***Y*** is the decomposition ***Y***=***UDV***^*t*^, where the columns of ***U*** and ***V*** are orthogonal, ***D*** is a diagonal matrix with nonnegative diagonal entries (singular values) presented in descending order, and the superscript *t* indicates a matrix or vector transpose. The sign of the dominant singular vector ***u***_1_ (the first column of ***U***) is fixed by requiring a positive average correlation with the columns of ***Y***; ***u***_1_ is referred to as the module eigenvector or eigengene. One can show that ***u***_1_ is an eigenvector of the *m*×*m* sample correlation matrix $\frac{1}{m}YY^{t}$ corresponding to the largest eigenvalue. The eigenvector ***u***_1_ explains the maximum amount of variation in the columns of ***Y***.

Let *d*_*i*_ be the *i*th singular value of ***Y***. The eigenvector factorizability

$$\begin{array}{ll} \text{EF}(u_{1})=\frac{|d_{1}{|}^{4}}{\underset{j}{\sum }|d_{j}{|}^{4}} & \; \end{array}$$

measures how well a network factors [18]. This measure is very similar to the proportion of variation explained, $d_{1}^{2}/\sum \overset{2}{\underset{j}{d}}$. One can prove [18] that when EF(*u*_1_) ≈ 1, the correlation matrix ***Y*** approximately factors as

$$\begin{array}{lll} \text{Corr}(x_{i},x_{j}) & \approx \text{Corr}(x_{i},u_{1})\text{Corr}(x_{j},u_{1}).\; & \; \end{array}$$

In co-expression networks, modules are often approximately factorizable [18,19]. For a network comprised of multiple modules, it should come as no surprise that when the eigenvector factorizabilities of all modules are close to 1, the correlation network factors as

(2) $$\;\begin{array}{l} A_{\text{ij}}\;\;\;\approx \;\;\;|\text{Corr}(x_{i},u_{1}^{c_{i}}){|}^{\beta }|\text{Corr}(x_{j},u_{1}^{c_{j}}){|}^{\beta }|\text{Corr}(u_{1}^{c_{i}},u_{1}^{c_{j}}){|}^{\beta }\\ \;\approx \;\;\;p_{i}p_{j}r_{c_{i}c_{j}}, \end{array}$$

where $u_{1}^{c_{i}}$ is the module eigenvector of the module containing *i*, $p_{i}=|\text{Corr}(x_{i},u_{1}^{c_{i}}){|}^{\beta }$ measures the intramodular connectivity (module membership) of node *i* with respect to its module, and $r_{c_{i}c_{j}}=|\text{Corr}(u_{1}^{c_{i}},u_{1}^{c_{j}}){|}^{\beta }$ measures the similarity between clusters *c*_*i*_ and *c*_*j*_. The quantity

(3) $$\begin{array}{ll} {\text{kME}}_{i} & =\text{Corr}(x_{i},u_{1}^{c_{i}})\; \end{array}$$

is called the module membership measure or conformity [18,19].

Unlike general networks, correlation networks allow assessment of the statistical significance of an edge (via a correlation test) and generate informative network statistics such as the module eigenvector. But correlation network methods can only be applied to model the correlations between numeric variables. An open question is whether correlation network methods can be generalized to general networks by defining a suitable decomposition of a general network similarity measure. In the following, we will address this question.

## Results and discussion

## CPBA is a sparse approximation of a similarity measure

Consider a general *n*×*n* symmetric adjacency matrix *A*, for example one generated by a multigraph. Because the diagonal entries of ***A*** are irrelevant, ***A*** is determined by its n2 upper-diagonal entries. We now describe a low-rank matrix approximation to ***A*** based on partitioning the *n* nodes into *K* clusters labeled 1,…,*K*. Motivated by (Eq. 2), our approximation of a general similarity relies on three main ingredients. The first is a **cluster assignment** indicator ***c*** = (*c*_*i*_) whose entry *c*_*i*_ equals *a* when node *i* belongs to cluster *a*. The cluster label *a* = 0 is special and is reserved for singleton nodes outside any of the “proper” clusters. The clusters are required to be non-empty except for the improper cluster 0. The second ingredient is a *K*×*K***cluster similarity matrix *R*** = (*r*_*ab*_) whose entries quantify the relationships between clusters. The third and final ingredient is the **propensity vector *p*** = (*p*_*i*_) whose components quantify the tendency (propensity) of the various nodes to form edges. The goal of cluster and propensity based approximation (CPBA) is to construct an approximation to ***A*** by optimally choosing the cluster assignment indicator ***c***, the cluster similarity matrix ***R***, and the propensity vector ***p***. CPBA assumes that the adjacency matrix *A*_*ij*_ can be approximated as

(4) $$\begin{array}{ll} A_{\text{ij}} & \approx r_{c_{i}c_{j}}p_{i}p_{j}.\; \end{array}$$

The right-hand side with K2+n parameters can be interpreted as a sparse parametrization of the left-hand side with n2 parameters. In a weighted correlation network, the propensity *p*_*i*_ of node *i* is approximately |kME_*i*_|^*β*^. The cluster similarity *r*_*ab*_, defined by the correlation $|\text{Corr}(u_{1}^{a},u_{1}^{b}){|}^{\beta }$ between eigengenes, is an intuitive measure of the interactions between modules. The diagonal entries *r*_*aa*_ of ***R*** are identically 1.

### Objective functions for estimating CPBA

In practice, CPBA parameters ***c***, ***p***, and ***R*** of a general similarity are unknown and must be estimated by optimizing a suitably defined objective function. In this article, we describe estimation methods that are based on optimizing two superficially different objective functions. Our first objective is just the squared Frobenius matrix norm

(5) $$\begin{array}{ll} \text{Frobenius}(c,p,R) & =\underset{i}{\sum }\underset{j\neq i}{\sum }{\left(A_{\text{ij}}-r_{c_{i}c_{j}}p_{i}p_{j}\right)}^{2}.\; \end{array}$$

Our second objective is the Poisson log-likelihood

(6) $$\;\begin{array}{l} \text{Poisson}(c,p,R)\;=\underset{i}{\sum }\underset{j\neq i}{\sum }\ln\left[\frac{{\left(r_{c_{i}c_{j}}p_{i}p_{j}\right)}^{A_{\text{ij}}}e^{-r_{c_{i}c_{j}}p_{i}p_{j}}}{A_{\text{ij}}!}\right]\\ \;=\underset{i}{\sum }\underset{j\neq i}{\sum }\left[A_{\text{ij}}\ln(r_{c_{i}c_{j}}p_{i}p_{j})\;-\;r_{c_{i}c_{j}}p_{i}p_{j}\;-\;\ln(A_{\text{ij}}!)\right]. \end{array}$$

Our later multigraph example interprets Poisson(***c***,***p***,***R***) in this traditional sense. The functional form of the Poisson log-likelihood even applies when the *A*_*ij*_ are non-integer. The factorial *A*_*ij*_!, which is irrelevant to maximization in any case, can then be defined via the gamma function. In practice maximization of the Poisson log-likelihood and minimization of the Frobenius norm yield very similar numerical updates.

In the Methods section, we describe a powerful MM algorithm for optimizing the objective functions and estimating its parameters. We now pause and briefly describe a few major applications. First, the sparse parametrization can be used to derive relationships between network statistics; our previous research highlights this possibility [18,19]. For example, the connectivity index (Eq. 1) can be approximated by

(7) $$\begin{array}{ll} k_{i} & =\underset{j\neq i}{\sum }A_{\text{ij}}\;\;\;\approx \;\;\;p_{i}\underset{j\neq i}{\sum }r_{c_{i}c_{j}}p_{j}.\; \end{array}$$

Second, since our optimization algorithms also strive to choose the best cluster assignment indicator ***c***, they naturally give rise to clustering algorithms. Cluster reassignment is carried out node by node in a sequential fashion. For the sake of computationally efficiency, all parameters are fixed until node reassignment has stabilized. If parameters are updated as each node is visited, then the computational overhead seriously hinders analysis of networks with ten thousand nodes. Our limited experience suggests that more frequent re-estimation of parameters is less likely to end with an inferior optimal configuration. Hence, the tradeoff is complex.

Other major uses depend on the underlying model. In the Frobenius setting, the model can be used to generalize conformity-based decomposition of a network as shown in Example 2. In the Poisson log-likelihood setting, our model suggest a new clustering procedure. In contrast to other clustering procedures, the CPBA models provide a means of relating clusters to each other via the cluster similarities *r*_*ab*_. Furthermore, likelihood based objective functions permit statistical tests for assessing the significance of an edge. For example, in the multigraph model, the significance of the number of connections between two nodes can be ascertained by comparing the observed number of connections to the expected number of connections under the Poisson model. Finally, likelihood based objective functions provide a rational basis for estimating the number of clusters in a data set.

In the following three examples, we illustrate how to generalize a variety of network models to include clustering.

### Example 1: Generalizing the random multigraph model

We recently explored a random multigraph model [20] that allows multiple edges to form between two nodes and edges to form with different probabilities. Edges still form independently. Under the random multigraph model, each node *i* is assigned a propensity *p*_*i*_. The random number of edges between nodes *i* and *j* is then assumed to follow a Poisson distribution with mean *p*_*i*_*p*_*j*_. This model relies entirely on propensities and ignores cluster similarities. We will refer to it as the Pure Propensity Poisson Model (PPP) to avoid confusion with CPBA. Thus, the PPP log-likelihood is

(8) $$\;\begin{array}{l} \text{Pure Propensity Poisson}(p)\\ =\underset{i}{\sum }\underset{j\neq i}{\sum }\ln\left[\frac{{\left(p_{i}p_{j}\right)}^{A_{\text{ij}}}e^{-p_{i}p_{j}}}{A_{\text{ij}}!}\right]\\ =\underset{i}{\sum }\underset{j\neq i}{\sum }\left[A_{\text{ij}}\ln(p_{i}p_{j})-p_{i}p_{j}-\ln(A_{\text{ij}}!)\right]\\ =\underset{i}{\sum }\underset{j\neq i}{\sum }\left[n_{\text{ij}}\ln(p_{i}p_{j})-p_{i}p_{j}-\ln(n_{\text{ij}}!)\right], \end{array}$$

where *A*_*ij*_=*n*_*ij*_ is the number of edges between nodes *i* and *j*. While future work could explore alternatives to the Poisson distribution, it is attractive for several reasons. First, it is the simplest model that gives the requisite flexibility. Second, a Poisson random variable accurately approximates a sum of independent Bernoulli random variables. A binomial distribution also serves this purpose, but it imposes a hard upper bound on the number of successes. Third, the Poisson model is convenient mathematically since it yields nice MM updates in maximum likelihood estimation of the model parameters [20]. Fourth, a likelihood formulation permits testing for statistically significant connections between nodes.

Although the parametrization (Eq. 8) of PPP is flexible and computationally tractable, it ignores cluster formation. To address this limitation, we propose to exploit the CPBA parametrization. This extension is natural because many large multigraphs appear to be made up of smaller sub-networks, often referred to as modules, that are highly connected internally and only sparsely connected externally. For example, consider a co-authorship multigraph where an edge is placed between two scientists whenever they co-author an article. Scientists working at the same institution and in the same department tend to be highly connected. Similarly, scientists tend to collaborate with other scientists working on the same research topics. Cluster structure is also inherent in biology. For instance, genes often function in pathways, and proteins often cluster in evolutionary families. Thus, when a network exhibits clustering, the propensity to form connections within a cluster is usually higher than the propensity to form connections between clusters. This phenomenon cannot be modeled using our original PPP model [20] and provides the motivation for injecting cluster similarity into the multigraph model. Our hope is that the CPBA based multigraph model will better account for differences in intracluster and intercluster connections and lead to better identification of significant connections. In the absence of an explicit model for clustering, the PPP model is likely to falter on a dataset that exhibits clustering. The most likely result is a host of significant connections between nodes in the same cluster since they all exhibit more edges than expected by chance. These types of significant connections are often uninteresting. In the above mentioned co-authorship network, the cluster structure may reflect institutional affiliations. In this case, it may be more interesting to identify pairs of researchers who publish more (or less) than is expected based on their workplace location.

To keep the number of parameters to a minimum, the cluster similarity matrix ***R*** = (*r*_*ab*_) is assumed to be symmetric with a unit diagonal. Thus, our new random multigraph model, CPBA, adds just K2 parameters for *K* clusters. As previously postulated, the number of edges between nodes *i* and *j* in clusters *c*_*i*_ and *c*_*j*_ is Poisson distributed with mean $r_{c_{i}c_{j}}p_{i}p_{j}$.

### Example 2: Generalizing the conformity-based decomposition of a network

To demonstrate the value in our clustering model and tap into the wealth of data on weighted networks [21], we propose a clustering extension. Because weighted networks by definition have edge weights in [0,1], we drop the Poisson assumption and instead minimize the Frobenius criterion (Eq. 5). A major benefit of this model is that it generalizes the conformity-based decomposition of a network [21]. An adjacency matrix ***A*** = (*A*_*ij*_) is exactly factorizable if and only if there exists a vector ***f*** = (*f*_*i*_) with non-negative elements such that

(9) $$\begin{array}{ll} A_{\text{ij}}=f_{i}f_{j} & \; \end{array}$$

for all *i*≠*j*. In this setting, *f*_*i*_ is often called the **conformity** of node *i*. Although the term **factorizable network** was first proposed in [19], numerous examples of these types of networks can be found in the literature. A physical model for experimentally determined protein-protein interactions is exactly factorizable [22]. In that model, the **affinity***A*_*ij*_ between proteins *i* and *j* is the product of conformities *f*_*i*_ = exp( − *α*_*i*_), where *α*_*i*_ is the number of hydrophobic residues on protein *i*. Since it can be shown that ***f*** is uniquely defined if the network contains *n* ≥ 3 nodes and all *A*_*ij*_ > 0 [19,21], it is easy to see that the propensity vector matches the conformity vector, ***p*** = ***f***, when all *r*_*ab*_ = 1. Even when a network is not factorizable, our method can estimate conformities while simultaneously clustering the nodes into more factorizable modules. In addition, the entries of the cluster similarity matrix **R** can be interpreted as adjacencies between modules. Thus, the cluster similarity matrix represents a network whose nodes are networks themselves. In correlation network applications, we proposed a similar measure [23], and for gene networks we defined a measure of the probability of overlap between gene enrichment categories. Although these measures are useful in their respective contexts, they cannot be generalized to other networks. In contrast, by incorporating cluster similarity into our model, we have a standard way of calculating these measures for any type of network.

### MM algorithm and R software implementation

Our software implementation of CPBA is freely available in the R package PropClust. On a laptop with a 2.4 GHz i5 processor and 4 GB of RAM, PropClust can estimate the parameters for 1000 nodes for a given cluster assignment in 0.1 seconds. For 3000 nodes, the same analysis takes 1 second. In practice, initial clusters are never perfect and must be re-configured as well. PropClust adopts a block descent (or ascent) strategy that alternates cluster re-assignment and parameter re-estimation until clusters stabilize. Block descent takes under 10 rounds on average if initial cluster assignments are good. Note that all parameters are fixed in cluster re-assignment, and all clusters are fixed in parameter re-estimation. Furthermore, both steps decrease the value of the objective function. Early versions of PropClust re-estimated parameters as each node was moved. This tactic proved to be too computationally burdensome on large-scale problems despite its slightly better performance in finding optimal clusters.

Judicious choice of the initial clusters is realized by a divide-and-conquer strategy. First, hierarchical clustering coupled with dynamic branch cutting [24] is used to cluster nodes into manageable blocks of user-defined maximum size, for instance at most 1000 nodes each. Second, the CPBA algorithm is applied to each block to arrive at clusters within blocks. Our co-expression network application shows that this initialization procedure works well even in large data sets. Another way to accelerate clustering is exploit parallel processing in the MM algorithm. Parallelization of the MM algorithm is easily achieved since the parameters separate in the surrogate function and updating the propensities via (Eq. 12) and (Eq. 15) requires only the previous parameter values. Cluster re-assignment avoids continuous optimization altogether and is very fast.

### Simulated clusters in the Euclidean plane

**Figure 1:**
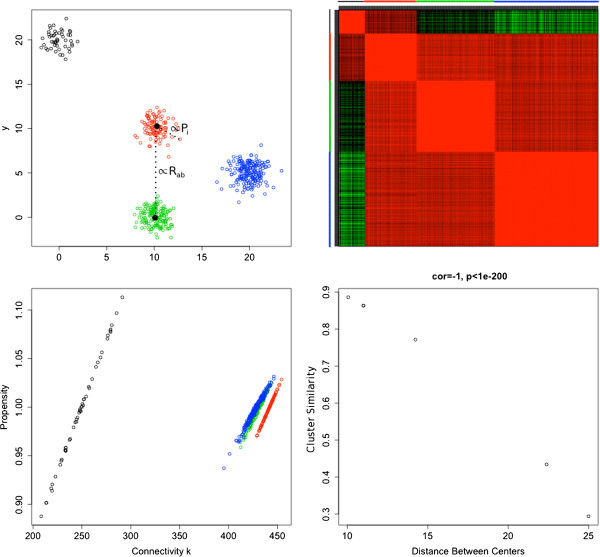
**Four clusters were simulated in the Euclidean plane by sampling from the rotationally symmetric normal distribution with means corresponding to the different cluster centers and variance matrix I.** The numbers of points in the clusters were 50, 100, 150, and 200 for the black, red, green, and blue clusters, respectively. **A**) A plot of the points is shown colored by cluster. **B**) Heatmap that color-codes the ordered adjacency matrix, calculated using the formula *A*(*i*,*j*) = 1 − [Euclidean.Distance(*i*,*j*)/ max(Euclidean.Distance(*i*,*j*))]^2^. In this plot red indicates a high adjacency, and green indicates a low adjacency. As expected, the adjacency within clusters is very high, and the adjacency between the blue and black clusters is the lowest since they are the furthest apart. **C**) The scatter plot between propensity (y-axis) and whole network connectivity (row sum of the adjacency matrix, Eq. 7) shows that the propensity is related to the distance between a point and its cluster’s center (given Eq. 10) in this example. **D**) Scatter plot between cluster similarity (y-axis) calculated using CPBA and the Euclidean distance between cluster centers (x-axis) shows a perfect negative correlation (-1).

Our first simulated dataset suggests a geometric interpretation of propensities and cluster similarities. For this dataset we simulated four distinct clusters on the Euclidean plane by sampling from a rotationally symmetric normal distribution with covariance matrix ***I*** and means corresponding to the four cluster centers shown in Figure 1A. The numbers of points in the four clusters were 50, 100, 150, and 200, respectively. The adjacency between two points is defined as 1−[dist / max(dist)]^2^, where dist denotes Euclidean distance between the points and max(dist) denotes the maximum distance between any two points. Thus as depicted in Figure 1B, points closer together have a higher adjacency than those further apart. As anticipated, the MM algorithm provided the correct cluster assignments. Figure 1C also makes it evident that the estimated propensity of a point is significantly correlated to the Euclidian distance between the point and its cluster’s center. This result is expected since a connectivity *k*_*i*_ is related to a propensity *p*_*i*_ through equation (Eq. 7). Within a module, connectivity is also related to its cluster’s center through the formula

(10) $$\begin{array}{ll} k_{i} & =(n-1)-\frac{n||x_{i}-\bar{x}|{|}^{2}+\underset{j}{\sum }||x_{j}-\bar{x}|{|}^{2}}{\max(\text{dist})}\; \end{array}$$

where *n* is the number of nodes in the cluster, ***x***_*i*_ is the position of node *i*, and $\bar{x}$ is the position of the cluster center [21]. This formula also explains why there is a separate line for each cluster in Figure 1C. Finally, Figure 1D shows that the cluster similarity *r*_*kl*_ of clusters *k* and *l* is significantly correlated to the distance between the centers of *k* and *l*. In summary, a propensity can be viewed as a measure of the centrality of a node, while a cluster similarity reflects the distance between two cluster centers.

### Simulated gene co-expression network

**Figure 2:**
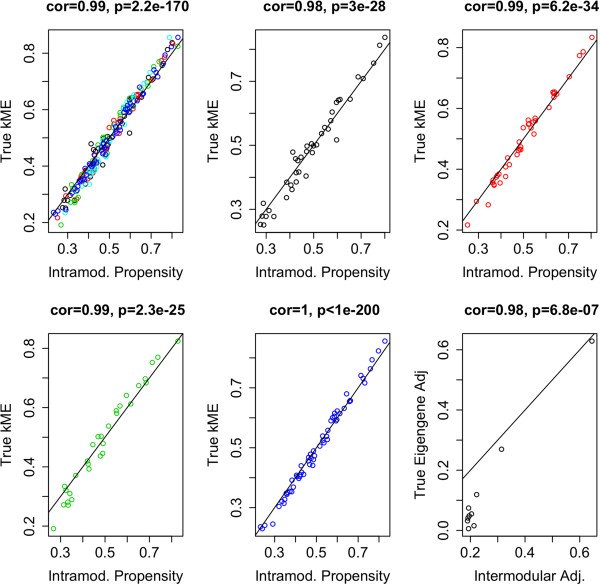
**Gene expression simulation results.** Gene expression data were simulated using the simulateDatExpr5Modules function under the WGCNA package in R. An adjacency matrix was then calculated from the Pearson correlation coefficients for the expression levels of each pair of genes. These plots reveal the relationship between the intramodular propensity and the true module membership, kME in (Eq. 3), first in all the clusters combined (top left) and then in each of the five clusters individually. Note the strong correlation and significant p-value in all cases.

To illustrate how CPBA generalizes to weighted correlation networks, we simulated gene expression data using the simulateDatExpr5Modules function in the WGCNA package in R [25]. Given the simulated expression data, we calculated Pearson’s correlation coefficient for each pair of genes and formed an adjacency matrix. Applying CPBA based clustering to the simulated data led to clusters that overlap perfectly with the simulated clusters. As Figure 2 depicts, the estimated propensities *p*_*i*_ are very significantly correlated to the node connectivities *k*_*i*_. This strong relationship reflects (Eq. 7). Furthermore, as seen in Figure 2, cluster similarity is significantly correlated to true eigengene adjacency, namely, $r_{c_{i}c_{j}}\approx |\text{Corr}(u_{1}^{c_{i}},u_{1}^{c_{j}}){|}^{\beta }$. In both simulations several different cluster assignment initializations were tried and all led to the same, correct, result.

### Real gene co-expression network application to brain data

**Figure 3:**
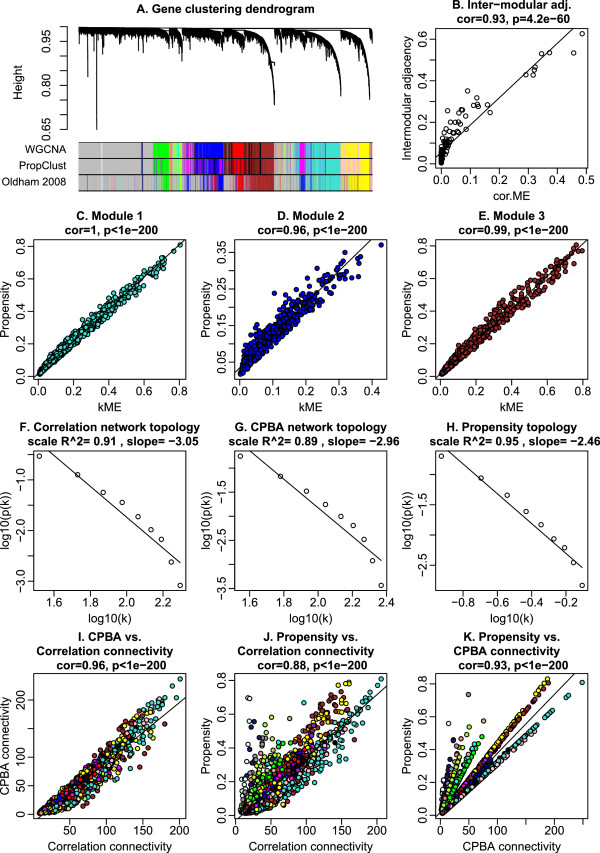
**Human brain expression data illustrate how CPBA can be interpreted as a generalization of WGCNA. A**) Hierarchical cluster tree based on WGCNA. Color bands show the WGCNA modules (first band), CPBA modules identified by propensity clustering (second band), and the modules identified by Oldham *et al*[9]. CPBA yields modules very similar to those identified by WGCNA. The overlap with the well annotated modules of Oldham *et al*[4] confirms that these clustering procedures yield meaningful modules. **B**) The intermodular adjacency calculated using CPBA (y-axis) is stronly correlated (*r* = 0.93) with its WGCNA counterpart, the correlation between eigengenes raised to the soft thresholding power. **C**) For nodes restricted to module 1 (turquoise in the color bands in panel **A**), CPBA propensity is highly correlated with its WGCNA counterpart, the module membership, kME (Eq. 3) raised to the soft thresholding power. **D**) and **E**) show analogous scatter plots for modules 2 (blue) and 3 (brown), respectively. **F**) The co-expression network exhibits approximate scale free topology (SFT). Specifically, the x-axis corresponds to equal width bins of the logarithm (base 10) of the connectivity $k_{i}=\underset{j\neq i}{\sum }A_{\text{ij}}$ (Eq. 1), and the y-axis reports the corresponding logarithm of the frequency. The approximate straight line relationship (linear model fitting index *R*^2^=0.91) indicates that SFT fits very well. **G**) evaluates SFT for CPBA connectivity defined by the right-hand side of Eq. 7. **H**) evaluates SFT for the propensity *p*_*i*_ only. **I**) The CPBA connectivity (y-axis) is highly correlated (*r* = 0.96) with connectivity *k*_*i*_ in the correlation network (x-axis). Genes are colored according to module assignment (PropClust color band in panel A. **J**) There is a high correlation (r = 0.88) between *k*_*i*_ (x-axis) and propensity (y-axis). **K**) There is a high correlation (r = 0.93) between CPBA based connectivity (x-axis) and propensity (y-axis).

In this real data example, we demonstrate that CPBA generalizes weighted correlation network analysis and can deal with fairly large data sets. The human brain expression data in question were measured on the Affymetrix U133A platform [4]. Following Oldham et al. 2008 [4], we restrict our analysis to the roughly 10^4^ probes that were highly expressed in brain tissue. The biological modules discovered by Oldham et al. 2008 [4] via WGCNA are fairly well understood and correspond to cell types such as astrocytes, oligodendrocytes, and neurons enriched for specific cell markers. In re-analyzing these data, we defined initial clusters as sketched in our discussion of the R software implementation of CPBA. This strategy obviates the need to pre-specify the number of clusters present in a data set. The results of PropClust are depicted in the second color band of Figure 3A. Overall, we find that CPBA yields modules very similar to those identified by WGCNA. The overlap with the well annotated modules of Oldham et al. [4] shows that the two clustering procedures yield meaningful and nearly equivlaent modules. CPBA has the advantage of giving cluster similarities. Figure 3B shows that eigengene based network adjacency (defined as the correlation between eigengenes raised to the soft-thresholding power 4) is highly correlated (r=0.93) with the cluster similarity parameter calculated by CPBA. For genes within a given module, Figures 3C-E demonstrate that the node propensities estimated under CPBA are highly correlated with the module membership measures *kME* raised to the soft thresholding power 4. Finally, Figures 3I-J show that the connectivities *k*_*i*_ in the correlation network are highly correlated (r=0.96) with the connectivities calculated under the CPBA approximation and with the corresponding CPBA propensities (r=0.88). Figure 3K shows that there is a high correlation (r=0.93) between CPBA based connectivity (i.e. the row sum of the CPBA matrix) and the propensity parameter.

These results demonstrate that CPBA is roughly equivalent to WGCNA in a typical co-expression network. We expect that CPBA will also be helpful in understanding network topology. For example, Figure 3F shows that the weighted co-expression network satisfies the approximate scale-free topology (SFT) property. Future research should aim to characterize the general fit of CPBA parameters to the SFT property. In this example, the CPBA based connectivities and propensities shown in Figures 3G and 3H agree well.

### OMIM disease and gene networks

Here we present an application that is not amenable to correlation network models but is arguably well suited for multigraph models. Specifically, we consider a bipartite multigraph between genes and diseases based on curated data from the reference Online Mendelian Inheritance of Man (OMIM), which tracks published links between diseases and corresponding genes [26]. These data were previously studied in detail by Goh et al. [27], who showed that diseases and their associated genes are related at higher levels of cellular and organ function. In the current application we validated their functional clustering using the CPBA model.

Following Goh et al. [27], we analyzed the data in two ways. First we created a disease network by placing an edge between two diseases for each gene they were both linked to. Only the links labeled as high quality by OMIM were considered. This construction yielded a multigraph of 2552 diseases with 1401 diseases connected to at least one other disease. We created a second, complementary multigraph by placing an edge between two genes for each disease they were both linked to. For this multigraph, there were 4045 genes with 1978 genes connected to at least one other gene. As suggested by the Medical Subject Headings (MeSH) list [28], we applied the CPBA model with *K*=10 clusters for the gene network and *K* = 14 clusters for the disease network, leaving out irrelevant categories.

**Figure 4:**
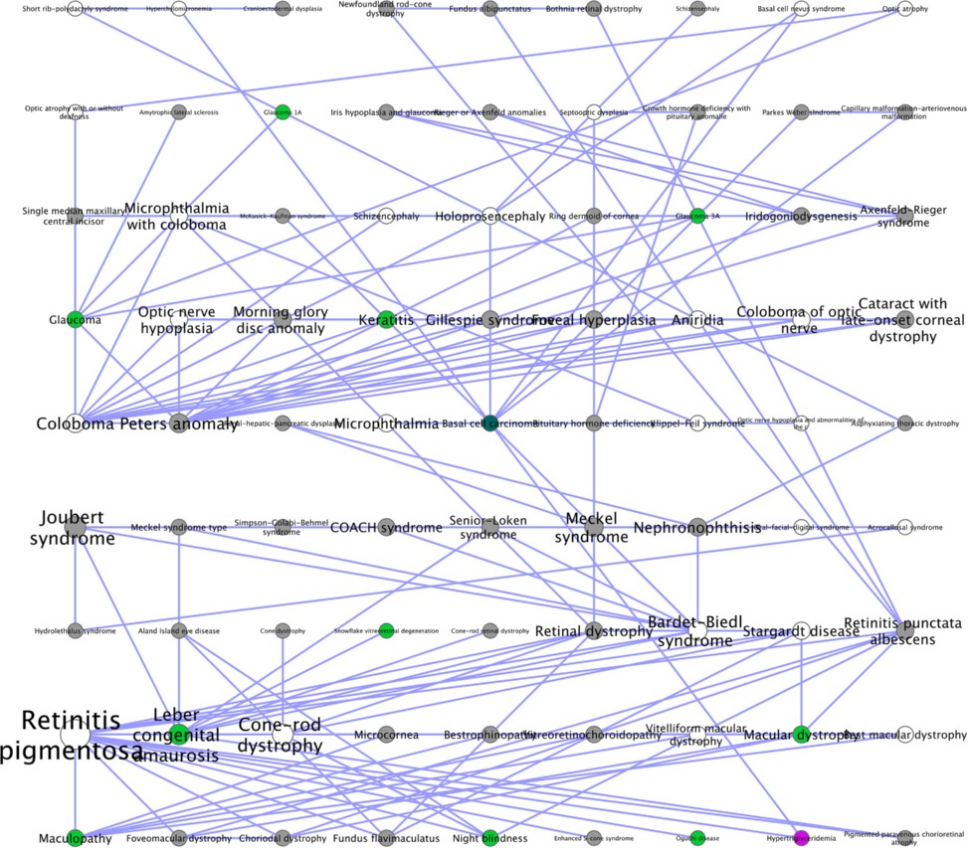
**OMIM disease network.** The intramodular connections between the nodes of the eye disease cluster are shown. Diseases are colored based on their MeSH categories, with diseases categorized as eye diseases (colored green), diseases linked to multiple categories (colored grey), and diseases that were not found (colored white). Note that more nodes should have been classified into the eye cluster by MeSH based on the name alone. Primary examples of this include retinitis pigmentosa, cone-rod dystrophy, retinal dystrophy, and microcornia. In spite of the failure of green labeling, these nodes were correctly classified by CPBA. Node and font sizes are proportional to a disease’s propensity.

We categorized the diseases using MeSH with little success. Nearly half of the diseases (47%) were not mapped to any category, and another 36% were mapped to multiple categories. Using the clustering obtained from the CPBA analysis of the disease network, we looked at whether any MeSH categories were overrepresented in a cluster. Ignoring diseases present in multiple MeSH categories, we found 8 significant categories at *P* < 0.01, including neoplasms, musculoskeletal diseases, and eye diseases (See Table 1). Although significant results were obtained, only a handful of diseases in each cluster contributed to the statistic. Upon closer inspection of the clusters, we found that many seemingly well-defined diseases were either not mapped or multiply mapped. For example, the eye disease cluster contains morning glory disc anomaly, coloboma, best macular dystrophy, cone-rod retinal dystrophy, and iris hypoplasia which are all clearly eye diseases, but not classified as such by MeSH. The eye disease cluster is depicted in Figure 4.

**Table 1:** Over-represented MeSH categories in the disease network

**Name**	**MeSH num.**	**−Log**_**10**_**(P)**
Hemic & lymphatic diseases	C15	8.32
Eye diseases	C11	7.78
Cardiovascular diseases	C14	4.23
Nervous system diseases	C10	4.04
Neoplasms	C4	3.37
Musculoskeletal diseases	C5	2.91
Endocrine system diseases	C19	2.04
Congenital, hereditary, &		
neonatal diseases & abnormalities	C16	2.03

Additionally, we found 540 significant connections between diseases at *P* < 0.01 and 148 significant connections at *P* < 0.001. The top 10 connections are listed in Table 2. The disease pair Adrenoleukodystrophy and Zellweger syndrome came in first; these two diseases are two of only three peroxisome biogenesis diseases belonging to the Zellweger spectrum [29]. It is also interesting to look for highly connected hub clusters, namely, clusters with high similarity to several other clusters. To define a measure of **cluster connectivity**, one can use the row sum of the cluster similarity matrix ***R***. The neoplasm cluster has the highest row sum and is the cluster with the highest cluster connectivity. This makes sense given the complexity and diversity of cancers within the cluster.

**Table 2:** Disease network top 20 significant connections CPBA

	**Disease 1**	**Disease 2**	**C1**	**C2**	**−Log**_**10**_**(P)**
1	Zellweger syndrome	Adrenoleukodystrophy	14	14	8.57
2	Muscular dystrophy-dystroglycanopathy (limb-girdle)	Muscular dystrophy-dystroglycanopathy (congenital)	2	2	7.05
3	Ullrich congenital muscular dystrophy	Bethlem myopathy	14	14	6.48
4	Iminoglycinuria	Hyperglycinuria	14	14	6.48
5	Alport syndrome	Hematuria	14	14	5.31
6	Colorblindness	Blue cone monochromacy	14	14	5.31
7	Refsum disease	Zellweger syndrome	14	14	5.05
8	Usher syndrome	Retinitis pigmentosa	8	6	5.04
9	Seckel syndrome	Microcephaly	14	14	4.96
10	Leukoencephalopathy with vanishing white matter	Ovarioleukodystrophy	14	14	4.96
11	Omenn syndrome	Severe combined immunodeficiency	14	14	4.90
12	Tuberous sclerosis	Lymphangioleio-myomatosis	14	14	4.60
13	Cone-rod dystrophy	Macular degeneration	6	10	4.60
14	Bronchiectasis with or without elevated sweat chloride	Pseudohypoaldoste-ronism	11	11	4.47
15	Leri-Weill dyschondrosteosis	Langer mesomelic dysplasia	14	14	4.10
16	Multiple pterygium syndrome	Myasthenic syndrome	14	14	4.00
17	Craniofacial-deafness-hand syndrome	Waardenburg syndrome	3	11	3.77
18	Nicotine addiction	Epilepsy	3	8	3.76
19	Hirschsprung disease	Pheochromocytoma	11	2	3.70
20	Langer mesomelic dysplasia	Short stature	14	14	3.62

**Figure 5:**
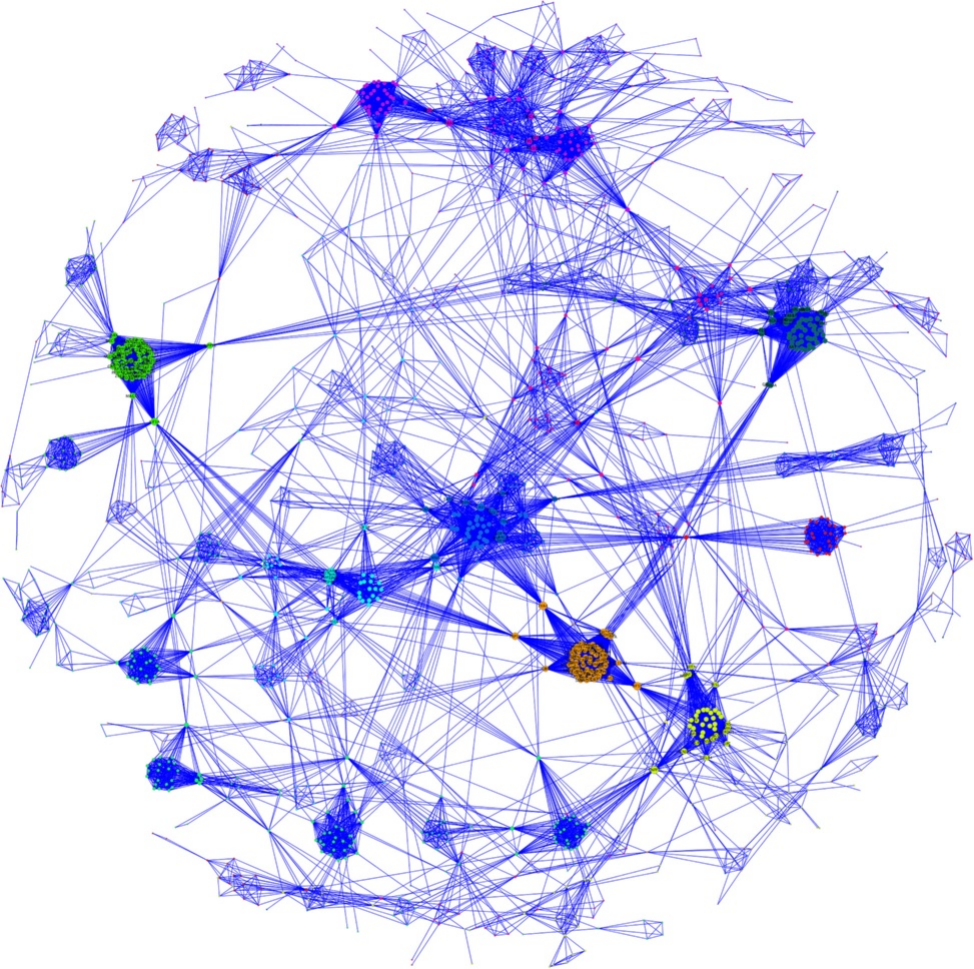
**OMIM Gene Network.** Genes are colored based on their cluster membership, and node size is proportional to a gene’s propensity. This view was achieved with a spring-embedded layout in Cytoscape using the number of edges between two genes as weights. Note that CPBA based clustering identifies modules of highly interconnected nodes.

Looking at the complementary gene network, we checked for overrepresentation of Gene Ontology (GO) terms using BinGO on Cytoscape [30]. We found that each cluster had an overrepresentation of many GO terms. In the cluster with the well-known tumor suppressor protein TP53, we found 875 statistically significant GO terms at *P* < 0.01. Of these, 585 terms are still significant at *P* < 0.001 after accounting for multiple testing. The top 10 GO terms include both positive and negative regulation of cellular and biological processes, regulation of cell proliferation, anatomical structure development, regulation of apoptosis, and others that are clearly associated with TP53. Finally, we found 1316 significant connections between genes at *P* < 0.01 and 418 at *P* < 0.001. The top 10 connections are listed in Table 3. Many of these gene pairs are known to interact from other supporting evidence. For example, interaction between the top ranking pair, Hemoglobin Alpha 1 globin chain (HBA1) and Hemoglobin Subunit Beta (HBB), is confirmed by their co-crystal structure in x-ray crystallography [31] and by automated yeast two-hybrid (Y2H) interaction mating [32]. Figure 5 depicts the full gene network derived from OMIM.

**Table 3:** Gene network top 20 significant connections CPBA

**Rank**	**Gene 1**	**Gene 2**	**Cluster 1**	**Cluster 2**	**- Log**_**10**_**(P)**
1	HBB	HBA1	2	2	9.05
2	SHOXY	SHOX	10	10	7.36
3	BDNF	HTR2A	5	4	7.07
4	SH2B3	JAK2	2	8	7.05
5	TSC2	TSC1	10	10	6.28
6	FOXC1	PITX2	7	7	5.73
7	MAPT	PSEN1	4	6	5.66
8	OPN1MW	OPN1LW	10	10	5.58
9	COL4A4	COL4A3	10	10	5.58
10	RAG2	RAG1	10	10	5.56
11	SCNN1G	SCNN1B	5	5	5.25
12	HBB	KLF1	2	10	5.09
13	COL6A1	COL6A3	10	10	5.08
14	COL6A2	COL6A3	10	10	5.08
15	SLC6A19	SLC36A2	10	10	5.08
16	SLC6A20	SLC36A2	10	10	5.08
17	SLC6A20	SLC6A19	10	10	5.08
18	COL6A2	COL6A1	10	10	5.08
19	GPC3	OFD1	8	7	4.75
20	LTBP2	CYP1B1	10	7	4.73

## Empirical comparison of edge statistics

In this section we compare our current CPBA model with our original Pure Propensity Poisson (PPP) model on two real datasets: the OMIM disease network and the complimentary OMIM gene network. On the whole we find that the CPBA model produces more plausible P-values for the edge-count tests. Conditioning on clusters enables CPBA to detect significant intercluster connections often missed by the PPP model. It also produces more reasonable P-values within clusters since propensities are not artificially deflated by the lack of connections between nodes from different clusters. We now consider how these trends play out in the OMIM disease network and the OMIM gene network.

In the disease network we find that, among the 20 most significant connections under the CPBA model, 5 are intercluster connections (See Table 2). Under the PPP model in contrast, none of the 20 most significant connections link different CPBA clusters (See Table 4). In fact, none of the top 50 connections of the PPP model occur between different CPBA clusters. The significant connection between Usher syndrome and retinitis pigmentosa would have gone completely unnoticed under the PPP model. This would be a shame because retinitis pigmentosa is a major symptom of Usher syndrome [33]. Another missed intercluster pairing, Waardenburg syndrome and Craniofacial-deafness-hand syndrome, also deserves recognition since both syndomes involve deafness and common facial features [34,35].

**Table 4:** Disease network top 20 significant connections PPP model

	**Disease 1**	**Disease 2**	**C1**	**C2**	**- Log**_**10**_**(P)**
1	Muscular dystrophy-dystroglycanopathy (limb-girdle)	Muscular dystrophy-dystroglycanopathy (congenital)	2	2	13.31
2	Zellweger syndrome	Adrenoleukodystrophy	14	14	12.06
3	Leber congenital amaurosis	Retinitis pigmentosa	6	6	10.12
4	Neuropathy	Charcot-Marie-Tooth disease	12	12	8.99
5	Blood group	Malaria	13	13	8.76
6	Ullrich congenital muscular dystrophy	Bethlem myopathy	14	14	8.57
7	Iminoglycinuria	Hyperglycinuria	14	14	8.57
8	Usher syndrome	Deafness	8	8	8.48
9	Hemolytic uremic syndrome	Macular degeneration	10	10	8.24
10	Bronchiectasis with or without elevated sweat chloride	Pseudohypoal-dosteronism	11	11	7.75
11	Refsum disease	Zellweger syndrome	14	14	7.14
12	Meckel syndrome	Joubert syndrome	6	6	7.08
13	Omenn syndrome	Severe combined immunodeficiency	14	14	6.99
14	Left ventricular noncompaction	Cardiomyopathy	12	12	6.97
15	Mitochondrial complex I deficiency	Leigh syndrome	2	2	6.85
16	Alport syndrome	Hematuria	14	14	6.70
17	Colorblindness	Blue cone monochromacy	14	14	6.70
18	Atrial fibrillation	Long QT syndrome	2	2	6.64
19	Cone-rod dystrophy	Retinitis pigmentosa	6	6	6.56
20	Microphthalmia with coloboma	Microphthalmia	6	6	6.46

Comparing the intracluster connections, we find that CPBA and PPP produce similar results, with 8 connections present in both lists. However, the P-values of these connections differ sharply under the two models. Since the PPP model essentially assumes a single cluster, estimated propensities trend lower in response to the lack of connections between nodes from different clusters. This results in lower means for the Poisson distributions and more extreme P-values. This phenomenon is especially evident in the pairing between Adrenoleukodystrophy and Zellweger syndrome; in the CPBA model the test for excess edges has −Log_10_(*P*) = 8.57, whereas in the PPP model −Log_10_(*P*) = 12.06.

The same story holds for the gene network. Among the 20 most significant connections under CPBA, 7 are intercluster connections (Table 3). Under the PPP model the corresponding number is 0 (Table 5). One of the more interesting missed connections occurs between BDNF (brain-derived neurotrophic factor) and HTR2A (seratonin receptor 2A). Both genes are associated with attention in schizophrenia [36]. As for intracluster connections, all intracluster connections found in the CPBA list are also found in the PPP list. However, the P-values for the most significant pair (HBB and HBA) differ by almost 5 orders of magnitude.

**Table 5:** Gene network top 20 significant connections PPP model

**Rank**	**Gene 1**	**Gene 2**	**Cluster 1**	**Cluster 2**	**- Log**_**10**_**(P)**
1	HBB	HBA1	2	2	13.87
2	SHOXY	SHOX	10	10	10.15
3	SDHD	SDHB	5	5	9.96
4	SCNN1G	SCNN1B	5	5	9.27
5	RAG2	RAG1	10	10	8.34
6	TSC2	TSC1	10	10	8.14
7	SDHC	SDHB	5	5	7.79
8	FOXC1	PITX2	7	7	7.54
9	OPN1MW	OPN1LW	10	10	7.43
10	COL4A4	COL4A3	10	10	7.43
11	GDF6	GDF3	7	7	7.29
12	TERC	TERT	9	9	7.20
13	CISH	TIRAP	4	4	7.12
14	GDNF	RET	5	5	7.04
15	COL6A1	COL6A3	10	10	6.94
16	COL6A2	COL6A3	10	10	6.94
17	SLC6A19	SLC36A2	10	10	6.94
18	SLC6A20	SLC36A2	10	10	6.94
19	SLC6A20	SLC6A19	10	10	6.94
20	COL6A2	COL6A1	10	10	6.94

**Figure 6:**
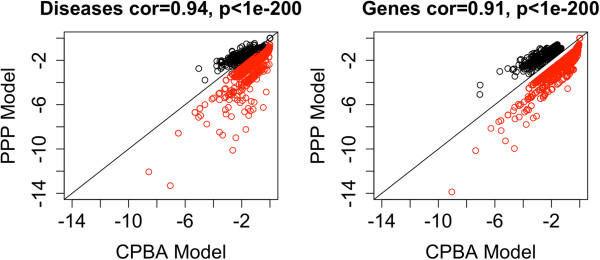
**OMIM CPBA versus PPP Analysis.** Scatterplot of the Log_10_(*P*) values obtained from analysis of OMIM using 14 and 10 clusters versus a single cluster for the Disease network and Gene network respectively. Note that the points are colored based on whether they come from a pair within a cluster(red) or between two clusters(black). This is very telling as it shows that by conditioning on the clustering, CPBA is able to increase its sensitivity in finding intercluster pairs while at the same time toning down that same trait in intracluster pairs.

To summarize, the CPBA model was able to find significant intercluster edge counts that the PPP model missed. Indeed, the PPP model was unable to find a single signficant intercluster pair in either data set. Although conditioning on clusters resulted in less impressive intracluster P-values, the CPBA model was still able to detect most of the significant intracluster pairings found by the PPP model. Figure 6 provides a scatterplot of −Log_10_(*P*) for all significant connections obtained under either model. Points are colored red if they represent a pairing within a cluster and black if they represent a pairing between different clusters. The figure justifies our contentions that the CPBA model is more sensitive to intercluster connections and less sensitive to intracluster connections than its less nuanced competitor. So while there will be fewer significant intracluster connections, they will arguably be more interesting. Most likely these virtues of the CPBA model carry over to other data sets.

## Simulations for evaluating edge statistics

**Figure 7:**
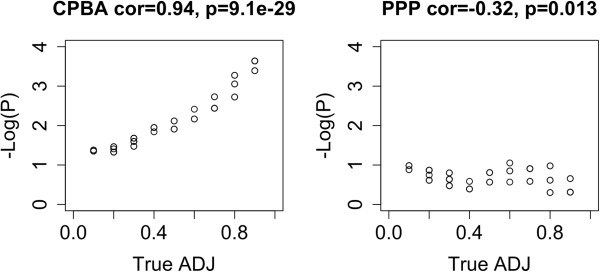
**Simulated CPBA versus PPP Analysis.** Scatterplot of the −Log_10_(*P*) values versus the true adjacency values obtained from 0/1 block diagonal matrix by re-setting a few other entries from 0 to 1. These changed values are shown along with the resulting −Log_10_(*P*) values obtained using CPBA and PPP.

To drive home the last point, we took a block diagonal adjacency matrix containing 1’s in its diagonal blocks and 0’s in its off-diagonal blocks and introduced a few off-block connections. In our initial matrix with three diagonal blocks of 100, 200, and 500 nodes, we changed 60 off-block entries from 0’s to 1’s. Each pair of node sets accounted for 20 of these switches. We then analyzed the modified matrix under both the CPBA and PPP models. Figure 7 plots −Log_10_(*P*) versus true adjacencies for the modified entries. Based on its identification of clusters, the CPBA model yields a better fit to the data. Comparison of −Log_10_(*P*) values under the two models shows that CPBA is more adept at finding significant intercluster connections. The evidence from the receiver operating characteristic (ROC) curve is very convincing on this point. The area under the ROC curve for the CPBA model was 0.95 compared to just 0.38 for the PPP model.

## Hidden relationships between fortune 500 companies

To illustrate the utility of CPBA in a non-biological setting, we briefly describe a multigraph model of cross-company management. Specifically, we took the Fortune 500 Companies of 2011 and put an edge between two companies for each shared member on their boards of directors. The original data is found in Freebase [37]. As discussed below, the use of the Bayesian Information criterion (BIC) or the Akaike Information criterion for estimating clusters is problematic. For example, the BIC suggests an optimal number of clusters *K* around 10, while the AIC gives a less plausible value of *K* > 20. In the following, we assume *K* = 10 clusters. It is noteworthy that most companies do not cluster into groups of related industries. This makes sense because conflict of interest norms preclude companies in the same field from sharing board members. Overt clustering is consequently discouraged.

Based on the underlying probability model, we ascertained the significance of the edge counts for company pairs. Table 6 lists the 10 most significant connections under the 10-cluster model. Several connections stand out. The significant pairing between Fidelity National Financial and Fidelity National Information Services is rather obvious. The same holds for the pairing between Autozone and AutoNation Inc. Other connections are less obvious. The pairing between General Motors and DuPont may reflect the fact that Pierre du Pont, the founder of DuPont, at one point owned a third of all General Motors stock. This remained true until federal antitrust prosecutors filed suit, and the Supreme Court ruled against DuPont, forcing the company to dispose all of its GM shares in 1961 [38]. Although the shares are gone, it seems that some ties persist.

**Table 6:** Fortune 500 top 10 significant connections

**Rank**	**Company 1**	**Company 2**	**- Log**_**10**_**(P)**	**Edges**
1	U.S. Bancorp	Ecolab	6.01	4
2	PetSmart	Dean Foods	4.53	3
3	Sempra Energy	Aecom Technology Corp.	4.39	3
4	General Motors	DuPont	4.07	3
5	Cardinal Health	Aon Corp.	4.07	3
6	Lockheed Martin	Monsanato	4.07	2
7	Fidelity National Financial	Fidelity National Inf. Services	4.06	2
8	Hewlett-Packard	News Corporation	3.89	2
9	AutoZone	AutoNation, Inc.	3.8	3
10	United Technologies Corporation	PACCAR	3.74	2

## Relationship to other network models and future research

Because so much effort has been devoted to the mathematical and statistical explication of complex networks, we can only touch on the relationship of the CPBA parametrization to other network models. Complex networks can be described by random graphs (the Erdös and Rényi model [39]), small-world models (the Watts Strogatz model [40]), scale-free networks (the preferential attachment model of Barabasi and Albert [41,42]), and other growing random network (GRN) models. These models involve graphs rather than multigraphs, so the number of edges per node pair equals 0 or 1. The CPBA has interesting ramifications for random graphs with arbitrary connectivity distributions [43]. If the edges are placed randomly in a network with such connectivities, then the probability *P*_*ij*_ of observing an edge between nodes *i* and *j* is exactly factorizable. In fact, $P_{\text{ij}}=k_{i}^{-3}k_{j}^{-3}$, where *k*_*i*_ is the connectivity (degree) of node *i*[42,44]. Thus, *P*_*ij*_ can be well approximated by CPBA with propensities $p_{i}=k_{i}^{-3}$ and cluster similarities *r*_*ab*_=1. The Erdös and Rényi (ER) model, which assumes uniform edge probabilities, is too restrictive for realistic networks. The CPBA parametrization adapts well to random graphs if we replace the mean edge count with the edge formation probabilities

$$\begin{array}{lll} P_{\text{ij}} & =\frac{p_{i}p_{j}r_{c_{i}c_{j}}}{1+p_{i}p_{j}r_{c_{i}c_{j}}}.\; & \; \end{array}$$

This reformulation of the model is consistent with construction of an MM algorithm for parameter estimation [45]. Future research should explore the topological properties of such models.

Growing random networks (GRNs) are also of interest since many networks grow by the continuous addition of new nodes and exhibit preferential attachment. Thus, the likelihood of connecting to a node depends on the node’s current connectivity [41-44,46,47]. At each time step of a growing random network [44], a new node is added, and a directed edge to one of the earlier nodes is created. This growing network has a directed-tree graph topology whose basic elements are nodes connected by directed edges. In general, the topology of a general GRN is determined by the connection kernel *A*_*k*_, which is the probability that a newly-introduced node forms an edge to an existing site with *k* edges (*k*−1 incoming and 1 outgoing). Future research could explore how to define a connection kernel (or more generally a GRN) so that the resulting network can be well approximated using the CPBA of the adjacency matrix. The Barabasi-Albert (BA) model is an important special case of a GRN [41,42] that leads to a scale-free network. In the BA model, the degree of a node satisfies the power-law (or scale-free) distribution

$$P(k)∼k^{\gamma }\;.$$

For homogeneous connection kernels, *A*_*k*_∼*k*^*ν*^, and scale free networks only arise if *ν* = 1 [44]. Future research could explore whether the adjacency matrix of the BA model can be well approximated using the CPBA. Toward this end it may useful to observe that the probability *P*_*ij*_ of finding an edge between nodes *i* and *j* in the BA model is given by [42,44]

$$\;\begin{array}{c} P_{\text{ij}}=\frac{4(k_{j}-1)(4k_{i}+k_{j}+2)}{k_{i}(k_{i}+1)(k_{i}+k_{j}-1)(k_{i}+k_{j})(k_{i}+k_{j}+1)(k_{i}+k_{j}+2)} \end{array}$$

which, importantly, assumes that node *i* with connectivity *k*_*i*_ was added later to the growing network than node *j* (implying that *k*_*i*_ <*k*_*j*_). In view of this temporal assumption, *P*_*ij*_ is not symmetrical in *i* and *j*; it also contains no parameters to capture clustering. Thus, there is no obvious relationship between the BA model and the CPBA approximation of a network. Future research can investigate how to parameterize preferential attachment so that the resulting probability *P*_*ij*_ of finding an edge fits well to the CPBA.

### Relationship to other clustering methods

Although the MM algorithm that estimates the CPBA parameters naturally generates a clustering method, CPBA is not just another clustering method. Our applications highlight the utility of the parameter estimates and the resulting likelihood based tests. CPBA not only provides a sparse parametrization of a general similarity matrix, but it also identifies hub nodes and clusters and enables significance tests for excess edges (between nodes) and shared similarities (between clusters). We do not claim that CPBA based clustering outperforms existing clustering methods in the simple task of clustering.

Substitutes for CPBA clustering include hierarchical clustering, partitioning around medoids [48], spectral clustering [49], mixture models [50], component models [51], and many more [52-56]. Because CPBA can be interpreted as a generalization of weighted correlation network methods, there is no need to invoke it instead of WGCNA when it comes to co-expression network applications. In modeling relationships between quantitative variables, one can use a host of other methods, for example sparse Gaussian graphical models [57,58], Bayesian networks, and structural equation models. CPBA is not meant to replace these powerful approaches for modeling relationships between quantitative variables. Its main attraction is that it applies to a general similarity measure. Since input data sometimes consists of a similarity (or dissimilarity) measure, CPBA fills a useful niche.

## Conclusions

The current paper introduces the CPBA model (cluster and propensity based approximation) for general similarity measures and sketches an efficient MM algorithm for estimation of the CPBA parameters. These advances will prove valuable in dissecting networks involving functional or evolutionary modules. The CPBA model is attractive for several reasons. First, it invokes relatively few parameters while providing sufficient flexibility for modeling observed similarities. Second, the cluster similarity parameters are good at revealing higher-order relationships between clusters. The row sum of the cluster similarity matrix can be used to define a cluster connectivity measure and to identify hub clusters such as the neoplasm hub in the disease network. Third, the CPBA model naturally generalizes network approximations that are already part of scientific practice, namely, the propensity based approach in multigraph models, the conformity decomposition in weighted networks, and the eigenvector based approximation in correlation networks. Fourth, the connections to the MM algorithm make the model well adapted to high-dimensional optimization. Fifth, the Poisson multigraph version of the model enables assessment of the statistical significance of edge counts and similarities between clusters. Sixth, likelihood-based models such as the Poisson multigraph model provide a rational basis for estimating the number of clusters. While it is beyond our scope to evaluate different methods for estimating the number of clusters in a data set, it is worth mentioning that our R implementation allows users to initialize clusters via hierarchical clustering. This tactic obviates the need to pre-specify the number of clusters.

Using simulated clusters in the plane and simulated co-expression networks, we demonstrate that CPBA generalizes existing methods. The planar examples show how a propensity can be intuitively seen as a measure of a node’s closeness to its cluster’s center and how a cluster similarity can be seen as a measure of proximity between two clusters. The simulated gene expression dataset exposes the CPBA model’s close ties to the previously studied concepts of intramodular connectivity, module eigengenes, and eigengene adjacency. Our analysis of real gene expression data reassures us that CPBA clustering results are similar to those of a benchmark method used in co-expression network analysis. The CPBA propensity parameters mirror the module eigengene based connectivity *kME*, and the cluster similarity measures mimic the network eigengenes. In our view, the main value of the CPBA model lies in generalizing correlation network methods.

To illustrate the versatility of CPBA, we applied it to the gene and disease networks of OMIM. The evidence that CPBA identifies biologically meaningful clusters is readily apparent in the significant enrichment of MeSH categories in the disease clusters and in the significant enrichment of GO terms in the gene clusters. While many other clustering procedures could have been used, CPBA has the advantage of dealing with dissimilarity measures as opposed to numeric input variables. It also provides Poisson likelihood based significance tests for edge counts (either pairs of genes and or pairs of diseases) that respect the underlying cluster structure. Finally, the row sums of the cluster similarity measure can be used to define hub clusters, and the estimated propensities can be used to define hub nodes. As we hoped, there were biologically meaningful ties between significantly connected pairs of genes and diseases. Several of these biologically plausible explanations are discussed in the text.

Although our examples are mainly biological, one can apply CPBA in many other contexts. For example, we employed CPBA to highlight shared board members among the Fortune 500 companies. This example illustrates how significant connections mirror the underlying ties between nodes. The edge count significance test suggests that the antitrust suit against GM and DuPont was no accident. To its credit, CPBA not only generalizes correlation network methods to general similarity matrices, but it also provides a valuable extension of random multigraph methods to weighted and unweighted multigraph data. CPBA is not just another clustering procedure but offers unique test statistics that permit identification of hub clusters and significant edge counts. We anticipate that the CPBA model will prove attractive to a wide range of scientists.

## Methods

### Maximizing the Poisson log-likelihood based objective function

Our algorithm for maximizing the Poisson log-likelihood (Eq. 6) given a clustering assignment ***c*** combines block ascent and the MM principle [59-61]. Clustering proceeds by re-assigning each node in turn until clusters stabilize. It may take several cycles through the nodes to achieve stability. Reassignment fixes parameters and selects the assignment with the highest log-likelihood. In the Poisson log-likelihood (Eq. 6), we take

$$\;\begin{array}{c} \ln L(c,R,P)=\underset{i}{\sum }\underset{j\neq i}{\sum }n_{\text{ij}}\left[\ln(r_{c_{i}c_{j}}p_{i}p_{j})-(r_{c_{i}c_{j}}p_{i}p_{j})-\ln(n_{\text{ij}}!)\right] \end{array}$$

where $r_{c_{i}c_{j}}$ is the cluster similarity between clusters *c*_*i*_ and *c*_*j*_, *p*_*i*_ is the propensity of node *i*, and *A*_*ij*_=*n*_*ij*_ is the number of connections between nodes *i* and *j*.

To optimize the objective function for a given cluster assignment, we employ block ascent and alternate updating ***R*** and ***p***. Fixing ***p***, it is possible to to solve for the best cluster similarity parameters *r*_*ab*_ exactly. Indeed, setting the partial derivative

$$\begin{array}{ll} \frac{\partial }{\partial r_{c_{i}c_{j}}}\text{Poisson}=\underset{k\in c_{i}}{\sum }\underset{l\in c_{j}}{\sum }\left(\frac{n_{\text{kl}}}{r_{c_{i}c_{j}}}-p_{k}p_{l}\right) & \; \end{array}$$

equal to zero and solving for $r_{c_{i}c_{j}}$ yields the simple update.

(11) $$\begin{array}{ll} {\hat{r}}_{c_{i}c_{j}} & =\frac{\underset{k\in c_{i}}{\sum }\underset{l\in c_{j}}{\sum }n_{\text{kl}}}{\left(\underset{k\in c_{i}}{\sum }p_{k})(\underset{l\in c_{j}}{\sum }p_{l}\right)}\;.\; \end{array}$$

We expect the estimated *r*_*ab*_ to occur within the unit interval [0,1] because edge formation is enhanced within clusters.

To update the propensity vector ***p*** with ***R*** fixed, we turn to an MM algorithm [59-61]. The MM principle says we should minorize the objective function by a surrogate function and maximize the surrogate function. This action drives the objective function uphill. One function minorizes another at a point ***p***_*m*_ if it is tangent to the other function at ***p***_*m*_ and falls below it elsewhere. The arithmetic-geometric mean inequality

$$\begin{array}{lll} p_{i}p_{j} & \leq \frac{1}{2}p_{\text{mi}}p_{\text{mj}}\left[{\left(\frac{p_{i}}{p_{\text{mi}}}\right)}^{2}+{\left(\frac{p_{j}}{p_{\text{mj}}}\right)}^{2}\right]\;\; & \; \end{array}$$

is the key to minorizing the Poisson log-likelihood. Substituting the right-hand side for *p*_*i*_*p*_*j*_ in the log-likelihood (Eq. 6) gives a surrogate function with parameters separated and leads directly to the propensity updates

(12) $$\begin{array}{ll} p_{m+1,i} & =\sqrt{\frac{p_{\text{mi}}\underset{j}{\sum }n_{\text{ij}}}{\underset{j\neq i}{\sum }r_{c_{i}c_{j}}p_{\text{mj}}}}\;.\; \end{array}$$

In practice, this MM algorithm may require an excessive number of iterations to converge. To accelerate convergence, we employ a Quasi-Newton extrapolation specifically designed for high-dimensional problems (Methods and [62]). The overall ascent algorithm (outer iterations) on ***R*** and ***p*** may also be slow to converge. It can also be accelerated by the same extrapolation scheme. Accelerating both inner and outer iterations gives a fast numerically stable procedure for estimating ***R*** and ***p*** for ***c*** fixed.

### Minimizing the Frobenius norm based objective function

Minimization of the Frobenius objective function (Eq. 5) employs block descent and again alternates updating ***R*** and ***p***. In this case setting the partial derivative

$$\begin{array}{ll} \frac{\partial }{\partial r_{c_{i}c_{j}}}\text{Frobenius}=-2\underset{k\in c_{i}}{\sum }\underset{l\in c_{j}}{\sum }(A_{\text{kl}}-r_{c_{i}c_{j}}p_{k}p_{l})p_{k}p_{l} & \; \end{array}$$

equal to zero and solving for $r_{c_{i}c_{j}}$ yields the simple update

(13) $$\begin{array}{ll} {\hat{r}}_{c_{i}c_{j}}=\frac{\underset{k\in c_{i}}{\sum }\underset{l\in c_{j}}{\sum }A_{\text{kl}}p_{k}p_{l}}{\left(\underset{k\in c_{i}}{\sum }p_{k}^{2})(\underset{l\in c_{j}}{\sum }p_{l}^{2}\right)}\;. & \; \end{array}$$

To update ***p*** for ***R*** fixed, we again rely on the MM principle. However, since we now seek to minimize the objective function, we majorize it. This is accomplished by first expanding it in the form

(14) $$\begin{array}{l} \text{Frobenius}(c,p,R)=\underset{i}{\sum }\underset{j\neq i}{\sum }\\ \;\times \left[A_{\text{ij}}^{2}-2A_{\text{ij}}r_{c_{i}c_{j}}p_{i}p_{j}+{\left(r_{c_{i}c_{j}}p_{i}p_{j}\right)}^{2}\right]\;. \end{array}$$

In majorization, one is allowed to work piecemeal. Thus, we majorize the term involving (*p*_*i*_*p*_*j*_)^2^ by the earlier arithmetic-geometric mean inequality

$$\begin{array}{lll} p_{i}^{2}p_{j}^{2} & \leq \frac{1}{2}{\left(p_{\text{mi}}\right)}^{2}{\left(p_{\text{mj}}\right)}^{2}\left[{\left(\frac{p_{i}}{p_{\text{mi}}}\right)}^{4}+{\left(\frac{p_{j}}{p_{\text{mj}}}\right)}^{4}\right]\; & \; \end{array}$$

taking into account squares. The term involving −*p*_*i*_*p*_*j*_ can be majorized by the inequality *x* ≥ 1+ ln*x* for *x* ≥ 0 in the form

$$\begin{array}{lll} -p_{i}p_{j} & \leq -p_{\text{mi}}p_{\text{mj}}\left[1+\ln\left(\frac{p_{i}p_{j}}{p_{\text{mi}}p_{\text{mj}}}\right)\right]\;.\; & \; \end{array}$$

Substituting upper bounds side for (*p*_*i*_*p*_*j*_)^2^ and −*p*_*i*_*p*_*j*_ in the expanded objective function (Eq. 14) gives a surrogate function with parameters separated and leads directly to the propensity updates

(15) $$\begin{array}{ll} p_{m+1,i}={\left[\frac{{\left(p_{\text{mi}}\right)}^{3}\underset{b}{\sum }\underset{j\in b}{\sum }r_{c_{i}c_{j}}A_{\text{ij}}p_{\text{mj}}}{\underset{b}{\sum }\underset{j\in b}{\sum }{\left(r_{c_{i}c_{j}}\right)}^{2}{\left(p_{\text{mj}}\right)}^{2}}\right]}^{1/4}\text{.} & \; \end{array}$$

As in the Poisson case, acceleration is advisable for both inner MM iterations and the outer block descent iterations. The same Quasi-Newton extrapolation [62] is pertinent and gives a fast numerically stable procedure for estimating ***R*** and ***p*** for ***c*** fixed.

### Model initialization

#### Initial cluster assignment

Many algorithms exist for creating initial cluster assignments [56]. For most datasets these assignments only affect the time to convergence and not the converged solution. Our R software implements hierarchical clustering and does not require pre-specifying the number of clusters. More specifically, our software applies average linkage hierarchical clustering with dynamic branch cutting [24]. Dissimilarities are set equal to 1 minus similarities.

#### Initial propensities

One way to initialize propensities is to assume a single cluster and estimate propensities as suggested in our earlier work [20]. An alternative in the Frobenius model is to initialize *p*_*i*_ by the sum of the connections of node *i* divided by the square root of the sum of all connections [21],

(16) $$\begin{array}{ll} p_{i}=\frac{\underset{j\neq i}{\sum }A_{\text{ij}}}{\sqrt{\underset{k}{\sum }\underset{j\neq k}{\sum }A_{\text{kj}}}}\;\text{.} & \; \end{array}$$

This initialization can be motivated by showing that the above equation holds if $r_{c_{i}c_{j}}=1$ (equivalently, the network consists of a single cluster) and $\sum p_{i}\gg \sum \overset{2}{\underset{i}{p}}$. While the assumption of perfect cluster similarity is unrealistic, it leads to initial values that work well in practice. For the Poisson model the analog is

(17) $$\begin{array}{ll} p_{i}=\frac{\underset{j\neq i}{\sum }n_{\text{ij}}}{\sqrt{\underset{k}{\sum }\underset{j\neq k}{\sum }n_{\text{kj}}}}\;\text{.} & \; \end{array}$$

#### Cluster similarity parameters

Because the block updates (Eq. 11) and (Eq. 13) for the cluster similarity parameters only depend on cluster assignment and propensities, it is natural to use those updates for initialization as well.

### Clustering algorithm

1. Choose the objective function (Frobenius or Poisson).

2. Initialize the cluster assignment, for example, via hierarchical clustering.

3. Initialize the propensity vector ***p*** by (Eq. 16) or (Eq. 17) and the cluster similarity matrix ***R*** by (Eq. 11) or (Eq. 13).

4. Parameter Estimation: Given cluster assignments, re-estimate parameters through the updates (Eq. 11) and (Eq. 12) or (Eq. 13) and (Eq. 15). Declare convergence when the objective function changes by less than a threshold, say 10^−5^.

5. Cluster Reassignment: 

(a) Randomly permute the nodes.

(b) For each node taken in order, try all possible cluster reassignments for the node.

(c) Assign the node to the cluster that leads to the biggest improvement in the objective function.

(d) Repeat step 5 until no nodes are reassigned.

6. Repeat steps 4 and 5 until no nodes are reassigned.

7. (Optional) Repeat steps 1- 5 for other cluster numbers and use a cluster number estimation procedure for choosing the number of clusters.

### Quasi-Newton acceleration

In this section we briefly review a Quasi-Netwon acceleration method described more fully in [62]. Newton’s method seeks a root of the equation **0** = ***x***−*F*(***x***), where *F*(***x***) is a smooth map. For CPBA this is the algorithm map summarized by Equations (11) and (12) for Poisson updates or Equations (13) and (15) for Frobenius updates. Because the function *G*(***x***) = ***x*** − *F*(***x***) has differential *dG*(***x***) = ***I*** − *dF*(***x***), Newton’s method iterates according to

$$\begin{array}{ll} \;x_{n+1}\;=\;x_{n}-\text{dG}{\left(x_{n}\right)}^{-1}G(x_{n})=x_{n}-{\left[I-\text{dF}(x_{n})\right]}^{-1}G(x_{n}). & \; \end{array}$$

Quasi-Netwon acceleration approximates *dF*(***x***_*n*_) by a low-rank matrix ***M*** and explicitly forms the inverse (***I*** − ***M***)^−1^.

Construction of ***M*** relies on secants. We can generate a secant by taking two iterates of the algorithm starting from the current iterate ***x***_*n*_. If we are close to the optimal point ***x***_*∞*_, then we have the linear approximation

$$\begin{array}{ll} F\circ F(x_{n})-F(x_{n})\approx M[F(x_{n})-x_{n}]\;,\; & \; \end{array}$$

where ***M*** = *dF*(***x***_*∞*_). We abbreviate the secant requirement as ***Mu*** = ***v***, where ***u*** = *F*(***x***_*n*_)−***x***_*n*_ and ***v*** = *F*∘*F*(***x***_*n*_)−*F*(***x***_*n*_). To improve the approximation of ***M***, one can use several secant constraints ***Mu***_*i*_ = ***v***_*i*_ for *i* = 1,…,*q*. These are expressed in matrix form as ***Mu*** = ***v***. For our purposes the value *q*=6 works well.

Provided ***U*** has full column rank *q*, the minimum of the strictly convex function $||M|{|}_{F}^{2}$ subject to the constraints ***Mu***=***v*** is attained at ***M***=***V***(***U***^*t*^***U***)^−1^***U***^*t*^[62]. Fortunately, a variant of Sherman-Morrison formula [63] implies that the matrix ***I***−***M*** = ***I***−***V***(***U***^*t*^***U***)^−1^***U***^*t*^ has the explicit inverse

$$\begin{array}{ll} {\left[I-V{\left(U^{t}U\right)}^{-1}U^{t}\right]}^{-1}=I+V{\left[U^{t}U-U^{t}V\right]}^{-1}U^{t}. & \; \end{array}$$

Thus, the quasi-Newton acceleration can be expressed as

$$\begin{array}{lll} x_{n+1} & =x_{n}-{\left[I-V{\left(U^{t}U\right)}^{-1}U^{t}\right]}^{-1}[x_{n}-F(x_{n})]\; & \;\\ & =x_{n}-[I+V{\left(U^{t}U-U^{t}V\right)}^{-1}U^{t}][x_{n}-Fx_{n})]\; & \;\\ & =F(x_{n})-V{\left(U^{t}U-U^{t}V\right)}^{-1}U^{t}[x_{n}-F(x_{n})].\; & \; \end{array}$$

This update involves inversion of the small *q* × *q* matrix ***U***^*t*^***U*** − ***U***^*t*^***V***; all other operations reduce to matrix times vector multiplications.

### Estimating the number of clusters

Estimating the number of clusters is the Achilles heel of cluster analysis. While this topic is beyond our scope, it is worth mentioning that an advantage of model based approaches is that likelihood criteria can be brought to bear. Since adding clusters entails more parameters, it is tempting to use the Akaike Information Criterion (AIC) or the Bayesian Information Criterion (BIC) to estimate the number of cluster in the Poissom model [64,65]. Both of these criteria balance the tradeoff between the number of parameters and the fit of the model. Specifically these methods choose the number of clusters *K* that minimize *AIC* = − 2 ln(*L*) + 2*c* or *BIC* = − 2 ln(*L*) + *c* ln(*n*), respectively, where *c* is the number of parameters, *L* is the likelihood, and *n* is the sample size. We caution the reader that AIC and BIC may be inappropriate for the present task because both criteria invoke strong assumptions. For example, AIC is derived by assuming a regular model, for instance, a linear model with Gaussian noise. Hence, AIC may be inappropriate for models with latent variables such as cluster labels. BIC may be inappropriate because our approach is frequentist rather than Bayesian. A review of the limitations and utility of these criteria can be found in [66].

### Ethical approval

This article involved publicly available human data sets which are completely anonymized. This study is therefore exempted from requiring ethics approval. No animal data were used. We fully comply with the Declaration of Helsinki and the “Animal Research: Reporting In Vivo Experiments” (ARRIVE) guidelines.

## Availability and requirements

**Project name:**PropClust R package **Project home page:**http://www.genetics.ucla.edu/labs/horvath/PropClust** Operating system(s):** Platform independent **Programming language:** R **Licence:** GNU GPL 3 The propensity based clustering method propensityDecomposition is implemented in the R package PropClust. The package also contains the function CPBAdecomp for carrying out the propensity decomposition of a network.

## Abbreviations

BA: Barabasi Albert; CPBA: Cluster and propensity based approximation; ER: Erdos renyi; GO: Gene ontology; GRN: Growing random network; kME: Connectivity based on the module eigenvector or eigengene; MeSH: Medical subject headings; MM: Minorization maximization or majorization minimization; PPP: Pure propensity poisson; SFT: Scale free topology.

## Competing interests

The authors declare that they have no conflict of interest.

## Authors’ contributions

JR, SH, and KL jointly developed the methods and wrote the article. JR carried out the analysis and implemented the software. PL helped with the R software implementation and carried out the example analysis on empirical expression data. All authors read and approved the final manuscript.
